# Evaluating genetic-based disease prediction approaches through simulation

**DOI:** 10.1007/s00439-025-02798-y

**Published:** 2026-01-21

**Authors:** Max Shpak, Eric Parfitt, Soroush Mahmoudiandehkordi, Mehdi Maadooliat, Steven J. Schrodi

**Affiliations:** 1 Department of Medical Genetics, School of Medicine and Public Health, University of Wisconsin-Madison, Madison, WI USA; 2 Wolfram Research, Inc., Champaign, IL USA; 3 Department of Mathematical and Statistical Sciences, Klingler College of Arts and Sciences Marquette University, Milwaukee, WI USA

## Abstract

Common diseases exhibit substantial heritability, and GWAS of these diseases have revealed hundreds of thousands of high-frequency disease susceptibility variants throughout the genome. These studies offer the prospect of using genomic data to improve disease prediction and diagnosis, however, the relative performance of different predictive modeling approaches is not well-characterized. To investigate this systematically, we constructed a Monte Carlo simulation generating model genomes with 500 SNPs carrying risk alleles that are parameterized by the strength of their effects and by different modes of inheritance—additive, dominant, recessive, and combinations thereof. After generating genotypes for cases and controls, several machine learning classifiers (logistic regression, naïve Bayes, random forests, and neural networks, with and without feature selection) were applied to predict disease phenotypes from genotypes. Each classifier’s error rates were evaluated and compared using AUC. We found that random forest models were the most accurate predictors of disease over the range of inheritance parameters, followed by logistic regression and naïve Bayes, while the feedforward multilayer neural network model had lower AUC. We also investigated the association of AUC with the difference in polygenic risk score (PRS) between disease and control samples by comparing AUC in the simulations to the values predicted from the PRS distributions, finding a monotonic, curvilinear relationship as predicted analytically from odds-risk and liability threshold models. Our results also show that with small risk effects, the odds-risk model provided an accurate estimate of the AUC-PRS association while a liability threshold model performed better when risk alleles had strong effects.

**Supplementary Information:**

The online version contains supplementary material available at 10.1007/s00439-025-02798-y.

## Introduction

Large-scale genetic association studies of complex diseases have markedly improved our understanding of the specific variants, genes, and pathways underlying a wide array of disease phenotypes and medically relevant traits (Beck et al. 2020; Visscher et al. 2017). These discoveries have played an instrumental role in (1) improving our understanding of inheritance patterns and genetic architecture of complex diseases (Watanabe et al. 2019), (2) providing insight into the roles of protein function, splicing, and gene regulation in disease (Maurano et al. 2012; Gallagher and Chen-Plotkin 2018), (3) identifying genetic signatures of therapeutic response (McInnes et al. 2021), (4) identifying drug targets and mechanisms (El-Husseini et al. 2020; Levey et al. 2021; Owen et al. 2010; Reay and Cairns 2021; Tachmazidou et al. 2019), and (5) have illuminated paths to better predict disease risk (Schrodi et al. 2014).

The use of genetic factors in disease prognosis is one of the key promises of modern human genomics. Identifying risk-predictive combinations of germline variants not only better assesses the likelihood that patients will develop a disease, it can also shift clinical diagnoses to earlier stages in the healthcare system, thereby providing opportunities for intervention prior to excessive morbidity. Early clinical applications of genetic information include neonatal screening programs (Baker et al. 2019; Farnaes et al. 2018), hereditary cancers (Burt and Neklason 2005; Samadder et al. 2020), and pharmacogenetics (Relling et al. 2019; Roden et al. 2018). Many of these initial efforts were understandably focused on monogenic traits with variants carrying very high effect sizes.

Researchers have also developed and implemented methods to combine signal from panels of disease susceptibility markers to predict complex diseases. One of the first statistically rigorous approaches to using an arbitrary number of disease-associated genetic markers for the purpose of prediction of disease traits was outlined by Yang et al. 2003. The approach used in their study was to calculate the likelihood ratio of the probability of a panel of multilocus genotypes in case (disease) vs. control (healthy) individuals. The likelihood ratio was estimated by a logistic regression model, enabling adjustment for covariates and interaction effects, which can then be used to calculate the positive and negative predictive values of disease using multilocus genotypes in genome-wide association studies (GWAS). Such analyses have identified large numbers of SNPs and other genomic markers correlated with polygenic diseases. At the lower end, studies identified approximately 100 SNPs in association with age-related macular degeneration (Klein et al. 2005; Fritsche et al. 2016), rheumatoid arthritis (Ishigaki et al. 2022), and non-familial Alzheimer’s (Andrews et al. 2023), while at the other extreme, 700–800 SNPs have been identified in GWAS for Type II diabetes (Iamamura and Maeda 2024) and asthma (Demenais et al. 2018).

Many GWAS of behavioral disorders and meta-analyses thereof have identified much higher numbers of associated SNPs, e.g. Ripke et al. (2014), Schizophrenia Working Group (2014) found > 8000 common SNPs associated with schizophrenia. This may reflect the heterogeneous nature of these conditions and aggregation of different syndromes with disparate underlying causes into single diagnoses. The fraction of null sites was (conservatively) selected to be somewhat higher than the standard false discovery rate (FDR) of 0.05.

Because of the large numbers of genomic predictor variables generated by GWAS, a number of disease risk metrics have been developed based on multilocus genotype effects—among the most widely adopted approaches is the construction of polygenic risk scores (PRS, also abbreviated as PGS in some of the literature), which are a weighted average of the per-SNP log-transformed odds ratios taken over a number of sites (Dudbridge 2013; Janssens et al. 2006; Purcell et al. 2009; Schizophrenia Working Group 2014). These metrics, which assume independent contributions across sites, have also been generalized and extended to include more realistic models of genetic architecture and to combine predictions from different analyses and classes of data.

Machine learning and deep learning algorithms are robust, powerful, and established methods for addressing classification problems, and offer alternatives to PRS-based methods of assessing disease risk from genotype data. Recently, machine learning tools have been applied to genetic data for the purpose of disease prediction (Mittag et al. 2015; Ho et al. 2019; Telenti et al. 2018; Wu et al. 2018). These methods include classic machine learning classifiers, such as random forests and support vector machines, as well as a variety of newer neural network-based algorithms such as convolutional and deep neural networks (Liu et al. 2022), generative adversarial networks, and self-organizing maps (e.g. Alzoubi et al. 2023; Bracher-Smith et al. 2021; Ghafouri-Fard et al. 2020; Patrick et al. 2018).

As genomic technologies advance and large-scale disease gene mapping studies continue to proliferate, the resulting wealth of genetic association data offers an opportunity to construct genetic-based and multi-omics-based predictive models for disease traits. However, little is known about the performance of these predictive models under different genetic architectures, or how these performances compare to other genotype-based disease risk assessment metrics. Allele frequencies, genotype penetrances, the number of susceptibility loci, and epistatic interactions all play a role in the genetic architecture of diseases. Understanding the impact of these parameters on the performance of various types of predictive modeling efforts is critically important for improving the accuracy of disease prediction from genomic profiles. For example, early work showed the importance of genotype frequency on discrimination accuracy in the context of type 2 diabetes (Janssens et al. 2007) and additional work examined the comparison between multiplicative and additive modes of inheritance (Moonesinghe et al. 2011).

It is plausible that different prediction algorithms have different strengths and weaknesses in their ability to capture disease susceptibility effects from combinations of SNPs, and that their relative predictive accuracies depend on the genetic architecture underlying disease risk. Understanding how the performance of various predictive methods changes as a function of disease genetic parameters will provide insight into which class of models will have the highest predictive accuracy for specific medical traits with disparate genetic architectures. The goal of this work is to explore the diagnostic utility of commonly used binary classifiers as a function of parameters for modeling disease genetics. With this aim, we developed a general disease multilocus genetic model which is implemented as a Monte Carlo simulation where we generate combinations of disease susceptibility SNPs/SNVs together with sites that are null with respect to disease and assess the efficacy of various classifier algorithms at predicting disease risk from genotypes.

Because patient risk is often evaluated using heuristic metrics, it is also important to assess the extent to which such metrics correlate with the accuracy of prediction models. Specifically, PRS have become a widely used approach to combine disease association signals from multiple susceptibility markers (Choi et al. 2020; see also De La Vega and Bustamante 2018; Jansenns 2019; Lewis and Vassos 2020 for discussions of applicability and limitations). PRS can also be used as a summary score to concurrently test for phenotype association against the combined effects of multiple SNPs, such as by refining PRS estimates by filtering predictive SNPs based on their p-values in GWAS statistics (Zhao et al. 2021, 2024). Polygenic risk estimation has been generalized to incorporate information from meta-analyses across multiple GWAS data sets to calculate a multi-PRS with higher predictive accuracy (e.g. Krapohl et al. 2018; Albinana et al. 2023) PRS has also been extended to models that incorporate information on linkage disequilibria among sites (e.g. Vilhjalmsson et al. 2015; Zhao et al. 2024) rather than assuming statistical independence and the excluding sites in LD.

Despite their extensive application, the efficacy of PRS as a predictor of disease incidence from genotype has not been systematically assessed through simulation, particularly not in the context of how differences in PRS between disease and control groups scale with the sensitivity and specificity of disease prediction under different classifier algorithms. One of the aims of this study is to assess the relationship between PRS values and predictive accuracy using simulated data, and to test analytical models of this relationship as derived in Dudbridge (2013) for log odds risk and threshold liability models.

## Materials and methods

### Disease model

The disease model employed simulated SNPs which varied in allele frequency and in the penetrances of their risk effects. This allows for modeling of both disease susceptibility SNPs and SNPs that are null with respect to disease status. Denoting alleles segregating at a modeled biallelic SNP as $\:{A}_{1}$ and $\:{A}_{2}$ and defining the genotype penetrances as $\:{f}_{ij}=P\left(Disease|{A}_{i}{A}_{j}\right)$, the frequency of the genotypes in the disease and non-disease populations is then.

1 $$\:P\left({A}_{i}{A}_{j}|Disease\right)={f}_{ij}\:P\left({A}_{i}{A}_{j}\right){\left[P\left(Disease\right)\right]}^{-1}$$

2 $$\:P\left({A}_{i}{A}_{j}|no\:Disease\right)=\left(1-{f}_{ij}\right)P\left({A}_{i}{A}_{j}\right){\left[1-P\left(Disease\right)\right]}^{-1},$$

respectively. The probability of disease will be the proportion of the disease trait attributable to the specific SNP, and calculated by $\:\sum\:{f}_{ij}P\left({A}_{i}{A}_{j}\right).$ Hardy-Weinberg equilibrium is assumed in the general population.

For the purposes of this study, we consider a simplified genetic architecture and ignore epistatic interactions, so that the contribution of each site to disease risk is statistically independent of other sites. This is often a reasonable approximation if the risk effects at each site are weak so that the second and higher order effects of epistasis can be ignored. Under this assumption of approximate additivity, the probability of a genotype conditional on disease (case) or control status at one locus is also independent of the genotype conditioned on disease or control at all other sites in the genome. The mode of inheritance is defined through simple relationships between penetrances of risk allele effects. Three standard models of inheritance were examined in the simulation: recessive, dominant, and additive. Although the penetrance relationships can depart from these simple models in complex diseases due to epistatic effects, evaluating these models serves as a step to explore the relative diagnostic utility across several types of genetic architecture. 

The recessive, dominant, and additive models are defined in terms of the penetrance relationships:

Recessive: $$\:{f}_{11}={f}_{12},\:{\:f}_{22}=\gamma\:{f}_{12}\:$$

Dominant: $$\:{f}_{22}={f}_{12}=\gamma\:{f}_{11}$$

Additive: $$\:{f}_{22}=\left(2\gamma\:-1\right){f}_{11},\:\:{f}_{12}=\gamma\:{f}_{11}$$,

where $\:\gamma\:>1$ is the genotypic relative risk (we do not simulate the case of $\:\gamma\:<1$, i.e. “protective” sites with alleles that reduce disease risk relative to a wild-type baseline in this study). In the additive model, the heterozygote is, by definition, exactly intermediate in disease risk between wild-type baseline and the genotype homozygous for risk alleles.

This general disease genetics framework has been used in numerous previous studies (Nielsen et al. 1998; Sham 1998; Schrodi et al. 2007; Maadooliat et al. 2016). We will also consider genetic architectures where the model genomes are composed of a mixture of effects across sites, i.e. the majority of sites with additive effects, and a smaller subset of sites with recessive and/or dominant effects in order to assess whether such mixed models were determined by the minority fraction of sites with dominant (or recessive) effects, versus being intermediate in comparison to additive and non-additive scenarios. Null genetic markers are modeled by setting all risk penetrances to a single constant value *f*, versus risk markers with variable perturbation term $\:\gamma\:$. 

All SNPs were generated in linkage equilibrium to model the effects from susceptibility and null loci randomly distributed across the genome. Both large-scale genetic studies and population genetics theory have shown that the site frequency spectra are accurately modeled by Beta densities, hence, the allele frequency at each site is sampled as a Beta variate (Ewens 1979; Gudmundsson et al. 2021). The two shape parameters for the Beta density ($\:\widehat{a}$ = 1.9195, $\:\widehat{b}$ = 5.6067 corresponding to an allele frequency expectation of 0.255 and variance = 0.0223) were estimated using the method of moments applied to all statistically significant SNPs from the GWAS Catalog (Buniello et al. 2019). Allele frequencies are assigned independently across sites in our model, in keeping with the assumption of approximate linkage equilibrium between pairs of sites.

### Polygenic risk score calculation

Polygenic Risk Score (PRS) calculation is an effective approach to capturing disease association across multiple genetic markers using a single univariate metric. A PRS is a weighted average of the effect size across genetic markers (e.g. SNPs or SNVs), that have been associated with a disease or some other binary phenotypic trait. Following Purcell et al. (2009), Schizophrenia Working Group (2014) and Polygenic Risk Score Task Force (2021), define the allelic PRS in the context of a dichotomous trait for an individual as

3 $$\:PRS=\frac{1}{2n}\sum\:_{i=1}^{n}ln\left({OR}_{i}\right){X}_{i},$$

where $\:n$ is the number of SNPs (sites) included in the PRS, $\:{OR}_{i}$ is the allelic case/control odds ratio for the $\:{i}^{th}$ SNP, and $\:{X}_{i}$ is the number of disease risk alleles that the individual carriers at the $\:{i}^{th}$ SNP (0, 1, or 2). 

In the definition above, $\:{OR}_{i}={f}_{cs}{\left({f}_{ct}\right)}^{-1}\left(1-{f}_{ct}\right){\left(1-{f}_{cs}\right)}^{-1}$; where $\:{f}_{cs}$ is the frequency of the disease risk allele at the $\:{i}^{th}$ SNP within cases and $\:{f}_{ct}$ is the frequency of that allele within the controls. The factor of 2 in the denominator accounts for diploidy and *max(X*_*i*_*)* = 2 at each site. There are other proposed heuristic metrics for PRS, such those with *OR* rather than *ln(OR)* in Eq. 3, but our study focuses exclusively on this definition, both because it is the most widely-used in the literature, and because this definition is congruent with the coefficients of a logistic predictor model of disease from genotypes (e.g. Wray et al. 2010; Dudbridge 2013).

### Monte Carlo simulation construction

To generate a large set of genetic markers with variable disease-susceptibility effects under models described above, we constructed a Monte Carlo simulation of disease risk in multilocus genotype samples. We simulated a scenario where risk-associated SNPs had been identified by GWAS, so that the majority of simulated sites carry risk alleles while a much smaller fraction are null sites (false positives in GWAS) which do not contribute to disease risk. We do not simulate the scenario of exploratory random assays of (potentially) millions of SNPs where the great majority would have null effects and only a very small fraction contribute to disease risk. All simulations were performed using R 4.3.0 and R 4.5.0, unless otherwise indicated.

Our experimental design for assessing the performance of classifiers in relation to inheritance model and penetrance $\:\gamma\:$ values involved simulating *n* = 2000 disease and 2000 control multilocus genomes in both the training and the test sets, so that the set of simulated genomes was balanced with respect to both disease/control and training/test set sample size (i.e. with a total of 4000 simulated genomes in both the training and test sets). Each genome in the simulation has L = 550 sites, consisting of 500 risk + 50 null polymorphic (biallelic) sites, i.e. where the former contribute to disease risk and the latter only to possible stochastic associations. The variable representing the diploid genotype at each site is an additive encoding, with a value equal to the number of risk alleles, i.e. 0 (no risk alleles – homozygous wildtype), 1 (heterozygote - single risk allele), or 2 (homozygous with two risk alleles), so that the model “genomes” are vectors of 0,1,2, and all prediction models are based on the association of disease with the number of risk alleles across sites. This additive encoding was chosen over multivariable encodings (e.g. binary variables for each allele or for each genotype) for its computational simplicity, and because previous studies, including Mittag et al. (2015), have shown that additive encoding provides predictive accuracy equivalent to or superior to multivariable encodings, at least in the absence of epistasis.

The total number of risk sites was chosen to be consistent (within an order of magnitude) with the number of sites identified in association with many common diseases by GWAS. The specific number of 500 was selected as a compromise between diseases with < 100 associated SNPs (e.g. macular degeneration) versus those with many hundreds (e.g. asthma), as summarized in the introduction.

The genotype at each site of a simulated genome is assigned by sampling from the conditional probabilities of genotype given disease or control phenotype in Eqs. 1–2, with the underlying assumption of Hardy-Weinberg equilibrium frequencies for the diploid genotypes at each locus together with a beta distribution parameterization of allele frequencies. Genotypes are assigned at each site independently of the alleles at all other loci, in keeping with the assumption of linkage equilibrium among all simulated loci. This assumption will be approximately valid if there are sufficiently many loci contributing to disease risk that their distribution across the genome is largely random, so that physical linkage across pairs of sites is rare (or if linked sites and those in regions of low recombination are specifically excluded in the model). It is also assumed that individuals represent a random sample from the population and do not share recent co-ancestry, so that the expected frequency of a risk allele at each site is approximately equal to its frequency in the general population.

We assign a baseline “wild type” disease rate *f*_*11*_ = 0.01, so that the probability of developing the disease in individuals with no risk alleles is 0.01. This baseline frequency includes the effects of non-genetic components to disease risk – i.e. environmental risk factors, on the assumption that genotype x environment interactions contributing to disease risk can be largely ignored. Deviations from this baseline due to the effects of risk alleles are determined by the penetrance parameter $\:\gamma\:=1+\:\delta\:$, defined above in the disease model, where the penetrance perturbation value $\:\delta\:$ is a uniform random variable on [0, 2 $\:\widehat{\delta\:}$], where *E[δ]* = $\:\widehat{\delta\:}$. We simulate genotypic risk over 16 values of $\:\widehat{\delta\:}$ ranging from 0 to 0.0075 in intervals of 0.005, with $\:\widehat{\delta\:}=\:$ 0 corresponding to the baseline risk of 0.01 for all genotypes and replicates while, for comparison, e.g. $\:\widehat{\delta\:}=$ 0.0075 generates disease risk as a uniform random variable in the range 0.01 to 0.025 (or, on average an expected 75% increase in disease risk in comparison to the baseline). The upper bound of 0.0075 was selected because in preliminary simulations, values of $\:\widehat{\delta\:}$ larger than this were associated with unrealistically high predictive accuracies (approaching 1.0) and thus provided little information.

The contributions of the perturbation values (measuring penetrance) to disease risk under additive, recessive, and dominant inheritance were parameterized as described in the previous section. In addition to modeling all 500 risk alleles as either completely additive, recessive, or dominant in their effects on disease phenotype, we also consider scenarios of mixed effects, i.e. 400 sites with additive effects + 100 sites with recessive alleles as well as the scenario of 400 additive + 100 dominant effect risk allele sites. These mixed models were introduced to determine whether the subset of sites with individually strong or weak effects would primarily determine the performance of classifier algorithms at predicting disease phenotype.

### Prediction modeling

From each iteration of the Monte Carlo simulation, the simulated multilocus genotypes composed of disease risk and null SNPs were used as features (predictor variables) for the development of machine learning models to predict disease status. The models were fitted using the training set with equal numbers of case and control genomes as described above. One set of analyses is carried out on a subset of sites filtered using LASSO (Tibshirani 1996) for feature selection, and a second set of analyses was run without LASSO. This approach allows a direct comparison of the performance of the classification machine-learning algorithms on LASSO-filtered data sets to those performed without prior feature selection. LASSO was implemented using the R 4.3.0 glmnet package (Friedman et al. 2010; Simon et al. 2011), selecting the penalty parameter λ from the range [0,0.05] in increments of 0.01. The feature selection had the additional constraint of requiring least 10% of the sites being retained for analysis – those that failed to return at least this fraction of sites were excluded from analysis.

All eight predictive combined scenarios (2 in the case of analyses with and without feature selection x 4 predictive models) were applied to data generated under a series of additive, dominant and recessive disease models (as well as the mixed inheritance models) to assess their binary classification performance. The predictive models used were: (i) *Logistic Regression* (*LR*, Hosmer and Lemeshow 1989), (ii) *Random Forest* (*RF*, Ho 1995), (iii) *Naïve Bayes* (*NB*, Domingos and Pazzani 1997) and (iv) *Neural Network* (*NN*, James et al. 2021; McCulloch and Pitts 1943) models to evaluate an array of different classifiers for the simulated data sets.

The RF and NB algorithms were implemented using the respective R packages randomForest (Liaw and Wiener 2002a, b) and e1071 (Meyer et al. 2024). We initially implemented neural network models using the R neuralnet library, but following a high failure rate in model convergence in preliminary analyses with < 10 replicates, we instead ran the h2o.deeplearning program (a feedforward multi-layer neural network, Candel et al. 2024), by interfacing R with h2o.ai (2022). The specific NN model used was a multilayer feedforward NN (FNN). This neural network model has the advantage of efficient processing speed and memory usage over models that encode sequential/temporal data, which are not relevant to the data structures analyze in this study. This, together with the hierarchical structure of a multilayer model, makes FNN being among the first and most widely-used neural networks applied to genotype-phenotype mapping in and biomedical genetics, e.g. Zafar et al. (2023).

Additionally, due to a failure of parameter estimates in some runs and a lack of consistency across other replicates with the standard R glm function, the h2o.glm function was used for predictive modeling with logistic regression. Unless otherwise noted, the default parameterizations and settings of each classifier in R and h2o were used to analyze the simulated genomes.

### Evaluation of AUC relationship to PRS

The simulated genotype – disease phenotype training and test set data were analyzed using LR, NB, RF, and NNs as described above. For each model, a tally of the number of true positives measured against the number of false positives is used to construct a Receiver Operating Characteristic (ROC) curve. The area under the curve (AUC) is a summary statistic of performance. The AUC can be interpreted as the probability that any two individuals, one with the disease and the other control, are correctly classified based on their genotypes. All AUC calculations were performed using the R pROC package (Robin et al. 2011).

The relationship between AUC and PRS was evaluated by computing the mean PRS within the case and control groups and assessing how ΔPRS = PRS_case_ – PRS_control_ (with a mean computed over all cases and controls per replicate) scales against AUC for a LR predictor. This ΔPRS vs. AUC relationship was assessed and comparisons were made across modes of inheritance with additive, recessive, and dominant effects (mixed effects were not considered for the PRS vs. AUC comparisons). The Pearson correlation between AUC and ΔPRS was also calculated to determine the strength of association between these summary statistics.

As part of our analysis of the relationship between predictive accuracy and the difference between case and control PRS in our simulated data, we compared the AUC observed for ΔPRS values generated in the simulations to the analytical predictions of AUC from the mean and variance of PRS in Dudbridge (2013). Dudbridge derived AUC for logistic regression models under two scenarios of additive genetic risk effects. The first scenario assumes that there is a hidden, normally distributed “liability variable” determined by additive effects across multiple loci. When this liability variable exceeds a threshold value (determined by the disease prevalence in the population and the assumption of normality of the liability variable), a disease phenotype results. Interpreting PRS as the coefficients of the liability function in a linear model, Dudbridge showed that the expected predictive accuracy given log odds PRS of the liability model is:

4 $$\:AUC=\:\phi\:\left(\frac{E\left[{PRS}_{case}\right]-E\left[{PRS}_{control}\right]}{\sqrt{Var\left[{PRS}_{case}\right]+Var\left[{PRS}_{control}\right]}}\right),$$

where $\:\phi\:$ is the cumulative density function of the standard normal distribution. For point of comparison with the simulations, our ΔPRS is a point estimator for the numerator term, the denominator is calculated from the observed variance in PRS across the 2000 case and control genomes.

Dudbridge also considered an alternative model where the PRS of an individual is the log risk of the disease for that genotype, rather than having PRS as an underlying liability variable with a threshold effect on disease risk. Assuming that the disease is rare in the population (i.e. background frequency < < 1) and equal variances in PRS for both case and control samples, the log-risk model has an AUC estimator of:

5 $$\:AUC=\:\phi\:\left(\sqrt{\frac{{R}_{PRS}^{2}{\left(1-K\right)}^{2}}{2P(1-P)}}\right),$$

where $\:{R}_{PRS}^{2}$ is the coefficient of determination of PRS on disease or control phenotype (computed from a simple linear regression of PRS in each replicate vs. binary disease phenotype using the *lm* function in R), *K* is the population prevalence of the disease (0.01 in our simulations), and *P* is the sample prevalence of the disease (0.5 in our model due to balanced sampling). In this study, we compare estimators of AUC in Eqs. 4–5 against the observed AUC under a logistic regression model for a range of PRS at different values of per-site genetic risk.

## Results

**Fig. 1:**
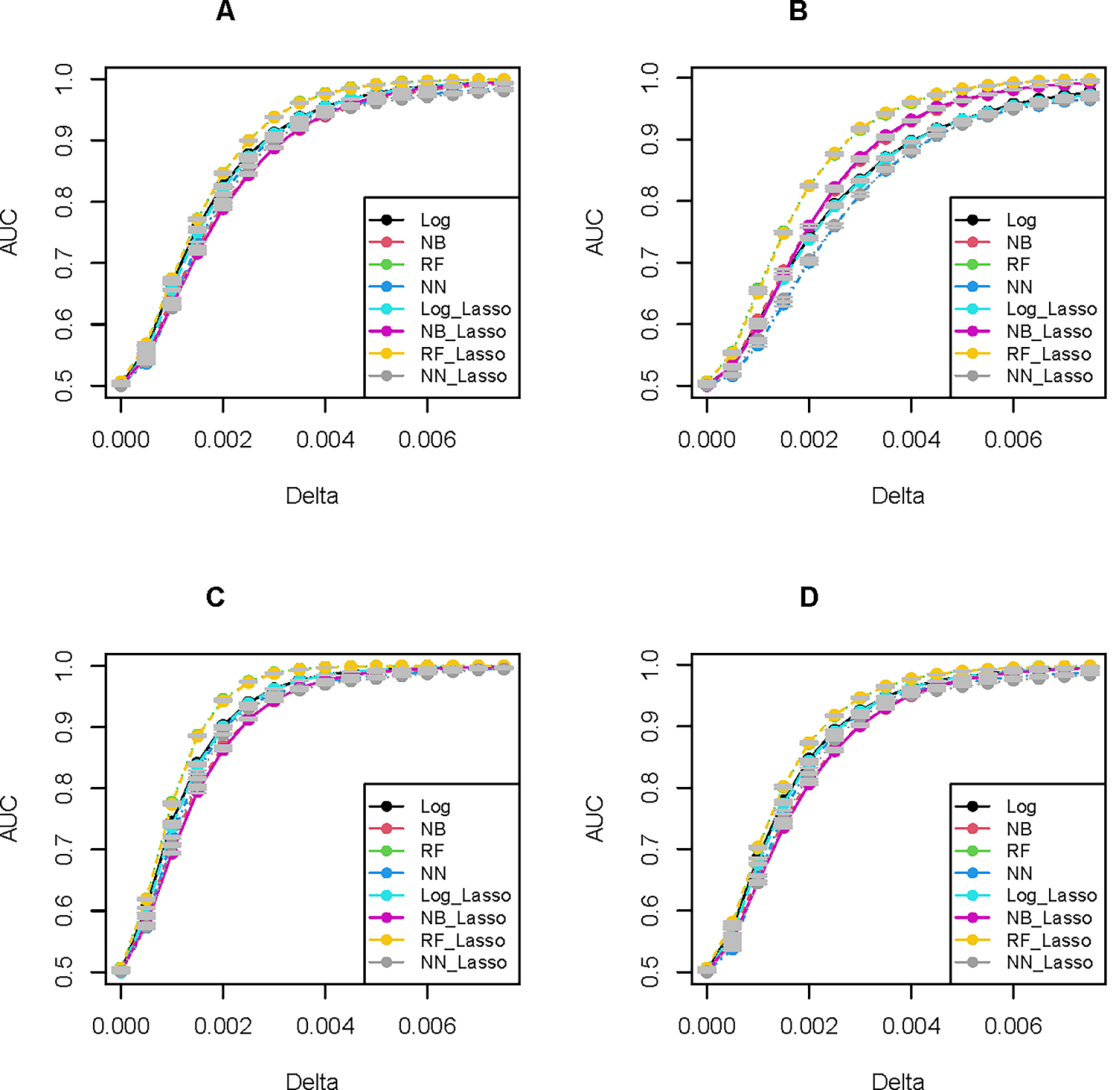
AUC vs. penetrance $\:\widehat{\delta\:}$ for various models of genetic risk inheritance across several predictive models (logistic regression, naïve Bayes, random forests, and neural networks), contrasting results with and without LASSO-based feature selection. Note that RF models give the highest predictive accuracy while NN give the lowest for most values, as well as the fact that feature selection has little impact on AUC. The AUC scores shown are the sample mean values over 100 replicates, together with confidence intervals of +/- 2 standard error units. **A** Additive model of disease risk inheritance. **B** Recessive model of disease risk inheritance. **C** Dominant disease risk inheritance. **D** Mixed additive-dominant model of disease risk inheritance (400 additive, 100 dominant effect sites).

**Fig. 2:**
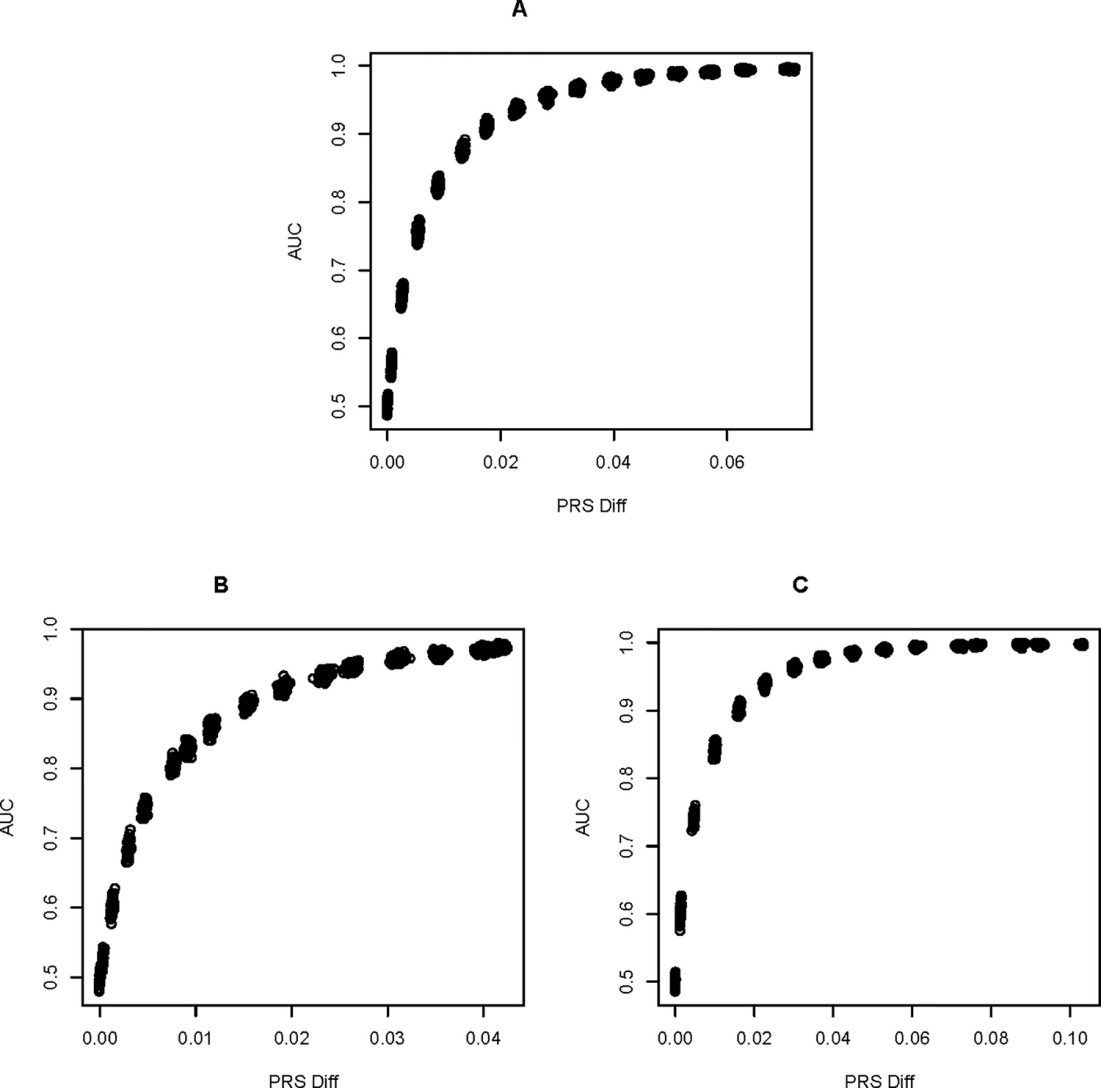
Plots of AUC vs. ΔPRS (the mean difference Polygenetic Risk Score between cases and control individuals) using logistic regression as a predictive model for **A** additive, **B** recessive, and **C** dominant models of disease risk inheritance, with all 100 replicates per $\:\widehat{\delta\:}\:$value included in the plots. The Pearson correlations between AUC and PRS are, for the three respective models of inheritance, 0.884 (additive), 0.924 (recessive), and 0.815 (dominant), with p < < 1e-6 for all scenarios.

There are several broad trends in our results concerning the association between penetrance values and AUC (Figs. 1 A-D, S1), and between AUC the heuristic PRS metric (Fig. 2A-C), which we summarize below.

### Comparison of classifier performance

As expected, AUC is effectively 0.5 for $\:\widehat{\delta\:}=0$ and monotonically increases with larger values of $\:\widehat{\delta\:}$ for all of the classifiers. Qualitatively, the general pattern of association between AUC and $\:\widehat{\delta\:}$ is one of approximately linear increase for values near zero before asymptotically approaching 1.0 for large penetrance $\:\widehat{\delta\:}$ values, resembling a sigmoidal curve. The fastest rate of increase in AUC with respect to penetrance (i.e. highest AUC for small penetrance values) occurs with dominant inheritance (e.g. with dominant allelic effects (Fig. 1C), AUC attains values ≥ 0.9 for $\:\widehat{\delta\:}=$ 0.002 with all classifiers apart from NN, whereas for additive (Fig. 1A) and recessive effects (Fig. 1B), this AUC value is not achieved for $\:\widehat{\delta\:}$ < 0.035 or in the range 0.0035–0.004, respectively, depending on the classifier).

For all modes of inheritance and over most of the range of penetrance $\:\widehat{\delta\:}$ values, RF classifiers out-perform all other algorithms in correctly predicting disease status from genotype, particularly at intermediate $\:\widehat{\delta\:}$ values. The greater predictive accuracy of RF in comparison to the other classifiers is especially pronounced with recessive effects of risk alleles, whereas for dominant effects the divergence of AUC among predictors is comparatively small. In contrast, the NN almost invariably had lower predictive accuracy when compared to the other predictive models – again with the greatest differences in performance in comparison to other classifiers observed with recessive inheritance. LR and NB are intermediate in performance between RF and NN. LR and NB give quite similar AUC values for additive and dominant inheritance, but in the case of recessive inheritance, they start to diverge for larger values of $\:\widehat{\delta\:}$, with LR consistently outperforming NB.

There is little improvement in classifier performance when including LASSO feature selection. The corresponding AUC values are nearly identical, as seen in Fig. 1, where most of the AUC averages with or without feature selection are almost superimposed. Indeed, in many instances the mean AUC is slightly larger in the absence of LASSO (e.g. with additive inheritance, the mean difference in AUC for LR with and without LASSO is 0.002, although this difference is not statistically significant, and, in contrast, the difference in mean AUC for NN under recessive inheritance with vs. without LASSO is −0.002, which is also not statistically significant). These results presumably reflect the fact that only 10% of sites in the model are null, so that feature selection may remove risk sites as false negatives along with the true negative nulls for models with weak risk effects, while for larger penetrance values, the classifiers are unlikely to fail in capturing effects from risk sites regardless of feature selection.

The AUC values for mixed-effect models, are, as expected, intermediate in comparison to pure additive and pure recessive or dominant, but more closely resemble the additive models (i.e. the majority of sites). For example, if we compare the AUC for the RF classifier in the mixed additive + dominant model (Fig. 1D), we can see that the AUC attains a value > 0.9 at $\:\widehat{\delta\:}\:$values between 0.002 and 0.0025, which is larger than the $\:\widehat{\delta\:}=0.0015$ for the case where all risk alleles have dominant effects (Fig. 1C) but slightly lower than the $\:\widehat{\delta\:}=0.025$ for the pure additive model. A similar intermediate pattern, though in the reverse direction, is observed for a mixed additive + recessive model (SI1). The AUC for mixed models more closely resembles the results for the pure additive effects than either the pure dominant or recessive effects, suggesting that the majority of sites in our model (400 out of 500 risk sites) with additive effects largely drive the predictive models and that the small subset of sites with non-additive inheritance effects do not appear to be the principal determinants of model performance, even when the non-additive minority have dominant effects.

The variance in AUC across replicates is quite small for all of the predictive models, as can be seen from the very narrow confidence intervals of +/- 2 standard error units around each point estimate in Fig. 1, e.g. the standard error of AUC (which for our sample size of 100 is 1/10 the standard deviation) values are of the order ~ 10^− 4^, e.g. ranging from 1.16e-4 to 8.48e-4 for logistic regression models with additive inheritance and values of similar size for other predictive models. These standard errors are orders of magnitude smaller than the increments of ~ 0.01–0.1 in the mean AUC across different values of $\:\widehat{\delta\:}$, and there is no overlap between the confidence intervals among the prediction models for most values of $\:\widehat{\delta\:}\:$(except in the trivial case of $\:\widehat{\delta\:}=0$, where all AUC are approximately 0.5 regardless of the simulated mode of inheritance).

### Predicting AUC from relative PRS

The plots of AUC vs. ΔPRS for the same $\:\widehat{\delta\:}\:$ are consistent with PRS being a strong heuristic predictor of disease risk and of classifier performance for our genetic models. Specifically, there is a significant correlation between ΔPRS and the AUC classifier performance metric for all models of inheritance, reflecting the close correspondence between ΔPRS and AUC for each increment in penetrance value (Fig. 2A-C for additive, recessive, and dominant inheritance—the dense clusters of points at regular intervals of the plot are the 100 replicates per penetrance increment).

As predicted, ΔPRS increases monotonically with $\:\widehat{\delta\:}$ as does AUC, thus the correlations between ΔPRS and AUC are statistically significant despite the non-linearity and asymptotic convergence to 1.0. Specifically, the Pearson correlations range from > 0.88 for additive and recessive effects to > 0.81 for dominant inheritance, with p < < 1e-6 for the correlation coefficients in all models. The faster rate of convergence to an AUC of 1.0 for dominant inheritance reduces the approximately linear component of the AUC vs. ΔPRS relationship and accounts for the somewhat smaller but still significant correlation coefficient. Note the larger values of ΔPRS for the same set of $\:\widehat{\delta\:}$ in the dominant inheritance model, i.e. a maximum < 0.05  in the recessive case vs. > 0.1 in the dominant effects case, indicating a greater divergence in disease vs. control odds ratios in association with dominant effect risk alleles.

**Fig. 3:**
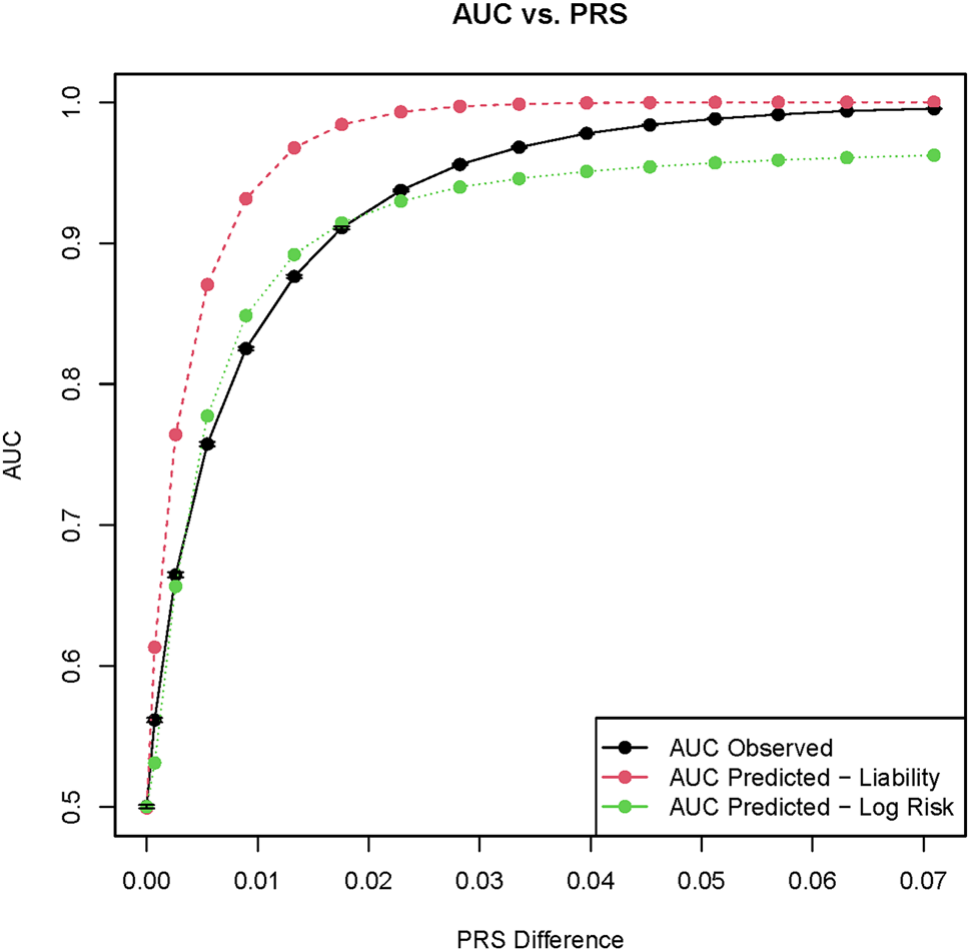
AUC vs. ΔPRS under a logistic regression predictor and an additive model of disease risk. The observed AUC (black) are similar to the predicted AUC (green) under a log risk model for smaller values of ΔPRS. The log risk predicted values (computed from R2 between PRS and phenotype) fail to converge to AUC values near 1.0 for larger deviations in PRS. In contrast, AUC predicted from PRS under a liability threshold model (red) is consistently higher than the AUC observed in our model, except asymptotically for very large ΔPRS. As in Fig. 1, confidence intervals of +/- standard error units are shown for the point estimates of the simulated AUC.

The relationship between mean AUC and mean ΔPRS (Fig. 3) shows a pattern consistent with that of a normal cdf as derived in Dudbridge (2013) for an additive model of disease risk inheritance, at least within certain ranges of ΔPRS. Figure 3 contrasts the observed relationship between predictive accuracy and case-control difference in PRS to the to the values of AUC predicted from the mean and variances of PRS in Eqs. 4–5. As expected, the variances in PRS among case and control are approximately normally distributed (p > > 0.05 for Kolmogorov-Smirnov test) and are nearly identical for small $\:\widehat{\delta\:}$, e.g. case and control variance in PRS = 1.81e-6 and 1.82e-6 at $\:\widehat{\delta\:}=0$. For large delta, the values are slightly divergent, e.g. for $\:\widehat{\delta\:}=0.05,$ the respective case and control PRS variances are 1.37e-4, 1.19e-4.

The AUC derived for a log-risk model for PRS (Eq. 5) provides a closer fit to the AUC vs. ΔPRS curve when the differences in PRS between case and control are small. However, for larger values of ΔPRS, the analytic approximation asymptotically tends to 0.96 rather than to 1.0 for the observed means and variances in PRS. In contrast, even though the liability threshold model (Eq. 4) generates a comparatively poor fit for small (but non-zero) values of ΔPRS and $\:\widehat{\delta\:}$, it does have the desired property of converging to 1.0 for the range of parameters considered.

Equations 4–5 were derived specifically for an additive model of disease risk, and thus would not precisely apply to scenarios with recessive or dominant effects. Nevertheless, as can be seen in the S2-A figures for dominant effects, the log-risk model still provides a relatively close approximation to the observed AUC when ΔPRS is small, while the liability threshold model AUC converges to the simulated values at larger ΔPRS values as in the additive case. Meanwhile, with recessive risk effects (S2-B), the log-risk model provides a better approximation for all ΔPRS values, because even at large ΔPRS the observed AUC converge to values < 1.0.

#### Summary and discussion

Disease genetics modeling is an understudied approach for understanding the implications and limitations of GWAS analyses and their implications for disease diagnostics. Because there have been few systematic studies to determine which types of classifiers and feature selection algorithms performed well for different disease models, this study (1) provides a framework for conducting Monte Carlo simulations to better elucidate the performance of standard machine learning approaches to genetic-based prediction, (2) shows that, at least for this class of non-epistatic models of genetic architecture, random forests seemed to provide the highest predictive accuracy, while other classifier models such as logistic regression and naïve Bayes also effectively predicted disease phenotype from genotype under the various models of inheritance, and (3) provides evidence that PRS is a valuable predictive tool for quantifying disease risk from genetic data, as evidenced by the correlation between model AUC and the separation in average PRS between case and control genomes (even with non-additive allelic effects), supporting their use as a disease risk metric.

This study also found, unsurprisingly, that dominant effects of risk alleles have higher diagnostic utility signal compared to those with additive effects, which in turn give higher AUC for the same perturbation values than under a recessive model of inheritance. These results are also consistent with the greater ΔPRS between case and control seen with dominant inheritance, as well as with the fact that the highest divergence in classifier performance is seen with recessive inheritance, suggesting that while all classifiers can effectively identify the strong association with dominant effects, differences in classifier model accuracy start to become more apparent when the predictor effects are weaker and/or rarer. This runs counter to some heuristic arguments suggesting that sites with recessive effects can sometimes generate the highest genotype-based odds ratios between disease and control (e.g. aa for disease vs. AA or Aa control) on the grounds that genotypes associated with disease would be particularly rare and distinctive, as opposed to cases where risk alleles in the heterozygous state could be found in both case or control individuals.

The differences in AUC among the predictive models suggest that random forests tend to correctly create decision trees with informative sites at the top of the hierarchy, outperforming non-hierarchical approaches such as LR and NB that also assign divergent weights across sites, but do not prioritize the order of sites considered when generating the classification models. This result may reflect the lower sensitivity of RF to stochastic outliers than e.g. LR, NB, as well as the tendency of RF to reduce variance and implicitly regularize the models by averaging over multiple decision tree models (Mentch and Zhou 2020; Liu and Mazumder 2024).

Several factors may be contributing to the relatively poor performance of neural networks in comparison to the other classifiers in our simulations. The first is that NN models typically require larger training sets for weight estimates to converge than LR, NB and RF coefficient estimation. This is partly due to the number of parameter estimates (weights) for NN being a product of the number of inputs and the number of neurons per layer, whereas in e.g. LR, NB the number of parameters equals the number of inputs. Several numerical analyses (e.g. Alwosheel et al. 2018) have indicated that sample sizes of at least 50x the number of weights are required for multilayer NN models to converge. This suggests that a sample size of 4000 for the training set, given 550 sites, may not be sufficient for the weight estimates in NN to converge.

Additionally, because NN models generate identify associations among predictor variables, they may create a class of false positive predictors that are absent in LR, NB, and RFs. Because we specifically simulate genotypes in the absence any non-stochastic associations among allele frequencies or effects, NNs have the potential to include false positive associations among variables into predictive models (Finkelstein et al. 2020) in addition to individual sites, whereas e.g. LR and NB do not. Conversely, this also suggests that given sufficiently large sample sizes, NN may be more effective at generating predictive models when there are in fact significant covariances in allele frequency and effects among multiple sites due to linkage and epistatic effects.

The observation that feature selection via LASSO has a negligible contribution to predictive model performance, as seen from the fact that mean AUC with or without LASSO for the same classifier are nearly identical, was somewhat surprising. This probably largely reflects the fact that we model a scenario where null sites are small fraction (< 10%) of the total number of SNPs, so that the number of false positives such sites can contribute is negligible compared to the associations with informative sites (of course, it is also the case that while < 10% of sites are true nulls, because site effects are simulated from a random uniform distribution, a somewhat larger fraction will be effectively null because of realized near zero δ). Regardless, when building predictive models using SNPs identified by GWAS, the set of loci that potentially contribute false positive predictors will be small relative to the number of informative sites and thus the benefits of feature selection will be marginal, as seen in our simulations results.

In contrast, for a scenario where the majority of sites are null -- e.g. cases where the set of loci was part of an exploratory study rather than identified by a prior GWAS – feature selection would probably play a much more important role in efficiently generating accurate predictive models. AUC provides a straightforward metric for assessing the confounding effect of the potentially high rate of false positives generated by stochastic associations of disease traits with null sites, and it is likely that there would be much larger differences in the AUC between models that utilized LASSO or other feature selection as filters for sites used in predictive models compared to predictive models created without feature selection.

Additionally, our simulations provide further support to the use of PRS as an indicator of disease risk, due to the strong association between ΔPRS and AUC both with additive and recessive/dominant effects. Apart from the heuristic and intuitive appeal of PRS, its association with AUC in the case of additive genetic architectures under a LR predictive model was shown analytically in Dudbridge (2013). The good fit of AUC in Eq. 5 to our simulated values in the lower range of ΔPRS implies that with small $\:\widehat{\delta\:}$ and small divergence in PRS between disease and control individuals, a log risk interpretation of PRS is a good model for disease prediction. In contrast, the divergence between observed AUC and that predicted from the log risk model for larger values of $\:\widehat{\delta\:}$ and ΔPRS suggest that some of the log risk model assumptions start to break down, as seen in the model’s failure to asymptotically converge to AUC = 1.0. The variances in PRS for the case and control samples diverge for the larger ΔPRS, violating the assumptions in the derivation of Eq. 5 (furthermore, an inflated error rate increases the sample variances of PRS and reduces AUC). The closer fit of Eq. 4 to the simulated AUC for high ΔPRS implies that for larger risk effects, a liability threshold model may be a more effective approximation for disease risk. This distinction is also seen from our results for recessive risk alleles (S2) where again the log risk model in Eq. 5 more closely approximates the AUC observed for larger values of ΔPRS.

A likely reason for the log risk model being a better predictor of AUC for small $\:\widehat{\delta\:}$ while the liability model more closely approximates the simulated data for large $\:\widehat{\delta\:}$ is that for larger effects, the variance in genetic risk in the general population increases. This results in a sharper partitioning between disease and control phenotypes with respect to their genotypes and their respective numbers of risk alleles, thus approximating a threshold liability model. In contrast, when $\:\widehat{\delta\:}$ and the variance in genetic risk are small, the genetic distances between disease and control individuals tend to be small as well, resulting in considerable overlap in sets of risk alleles between disease and control genotypes. Under that scenario, the liability model with its sharp threshold effect is not a good approximation to AUC due to the high overlap in genotypes between case and control, while a log risk model would be more effective at predicting this less discretized disease incidence. We also note that while we only fit AUC vs. PRS models for LR because of the analytical relationship between PRS and logistic regression coefficients derived by Dudbridge, the shape of the curves for the associations between AUC and $\:\widehat{\delta\:}$ are consistent across other predictive models (e.g. RF, NB), and it can be surmised that the correlations between PRS and AUC would be significant and a good fit under other classes of predictive models.

While our results convincingly demonstrate the ability of commonly-used classifier algorithms to predict disease phenotype from risk allele genotypes for the simple genetic architectures considered here, there are several limitations to this initial study of predictive model performance evaluation across disease models, suggesting future directions with more complex and biologically realistic scenarios. An important extension involves simulating disease risk in samples of individuals across different ancestries, because populations from different ethnic groups or geographic regions are divergent in the number, identity, and effect of risk-associated SNPs as well as in the risk allele frequencies at these sites. GWAS research and biobank data today take into account geographic and ethnic variation by sampling and analyzing genomes of individuals with ancestry outside of northwestern Europe (e.g. Peterson et al. 2019; Fitipaldi and Franks 2023), thus providing information on genetic architecture and allele frequencies that can readily be accommodated into our model by changing the relevant parameters, such as the distribution of risk allele frequencies or the number of risk-associated loci.

A greater challenge to the applicability of the models used here comes from statistical associations among sites and their effects, as our simulations generate each SNP in linkage equilibrium with respect to all other sites. This is often a reasonable approximating assumption for modeling unique disease susceptibility loci across the genome, due to the fact that many GWAS studies identify sets of SNPs where the overwhelming majority have LD near 0, and because SNP panels generated from GWAS studies for PRS-based disease prediction typically only use a small fraction of sites with leading effects, ignoring second-order effects due to their associations with other sites. Our results should be applicable to the fairly wide class of genetic architectures where the disease phenotype is determined by large numbers of loci (so that most pairs will be located on different chromosomes or at sufficient distances on a chromosome for free recombination) and where the majority have individually weak effects, so that epistatic effects can largely be ignored. Furthermore, it has been standard practice in studies leveraging PRS to exclude sites in high LD, particularly those in low-recombination regions, e.g. Choi et al. 2020.

However, incorporation of linkage disequilibrium patterns and other correlational effects, such as epistatic interactions and LD contributions to disease risk, would further generalize the results, as it is known that significant LD occurs among many 10–100 kb regions of human chromosome, with D sometimes being much higher than predicted under neutral evolution despite LD near 0 for more distant sites (e.g. Collins et al. 1999; Pritchard and Przeworski 2001). The assumption of linkage equilibrium may have contributed to logistic regression and the naïve Bayes classifiers outperforming a neural network predictive model, which, unlike NB and LR does not explicitly assume conditional independence between features. On the other hand, if the underlying assumptions of statistical independence are violated by linkage disequilibria or by genetic architectures with strong epistatic components, one may expect that neural networks would outperform classifiers such as LR or NB because of their ability to incorporate joint associations among sites as part of their predictive models, as was noted above.

Introducing LD and epistasis also creates associations in risk effects across sites that violate the assumptions behind standard PRS calculations, for example, sites with very high LD will be effectively redundant from the standpoint of covariance with a phenotypic trait. In order to correctly estimate genetic contributions to disease risk and to assess the association between risk and the rate of false discovery (as estimated by AUC) in data sets where LD are prevalent, it will be necessary to apply generalizations of PRS that include corrections for correlations and effective redundancy among loci due to LD among sites (e.g. Viljjalmsson et al. 2015, Zhao et al. 2024). Furthermore, modeling epistasis and LD introduces a number of computational challenges, the most obvious being the need for much larger simulated sample sizes to capture second order interactions through combinations of genotypes at different loci. This is particularly true if sets of site pairs rather than individual sites are used as predictor variables, which increases model dimensionality quadratically. Thus, analyses of epistatic interactions may require not only larger sample sizes, but different modeling scenarios, including genotype encoding. For example, He and Parida (2016) found that target-based encoding of SNPs at different sites (where the three genotypes at locus *i* are potentially assigned different numerical values than the three genotypes at locus *j* to reflect their association with phenotypic classes), provide more accurate phenotype prediction than additive encoding at each locus in both regression and SVM models, particularly in the presence of epistasis.

In summary, while this study generated useful preliminary results for understanding the performance of different classifiers using disease genetic data, additional work that generalizes the disease models, expands the size of the study, and considers additional methods to discriminate predictive accuracy across models will further advance this line of research. Indeed, expanding the size of the simulations, both with respect to the number of individuals and the number of SNPs may improve the predictive accuracy of the models even with simple genetic architecture, such as when assessing the efficacy of neural networks as predictive models.

## Supplementary Information

Below is the link to the electronic supplementary material.

S1. AUC vs. penetrance $\:\widehat{\delta\:}$ for a mixed inheritance model (400 additive, 100 recessive effect sites). Colors are the same as for Fig. 1.

S2. AUC vs. PRS difference for dominant (A) and recessive (B) models of inheritance (in black), compared to predicted values of AUC from PRS values under an additive model of inheritance. As in Fig. 3, the green line shows predicted values with a log odds risk model, the red line predicted AUC with a liability threshold model.

## Data Availability

The R code for the Monte Carlo simulation is publicly available at https://github.com/mshpak76/Genetic_Disease_Simulations-/[].
