# Supplementary figures and images for: Evaluating genetic-based disease prediction approaches through simulation
Source: Hum Genet. 2026 Jan 21;145(1):14. doi: 10.1007/s00439-025-02798-y (PMC12823641; doi:10.1007/s00439-025-02798-y)

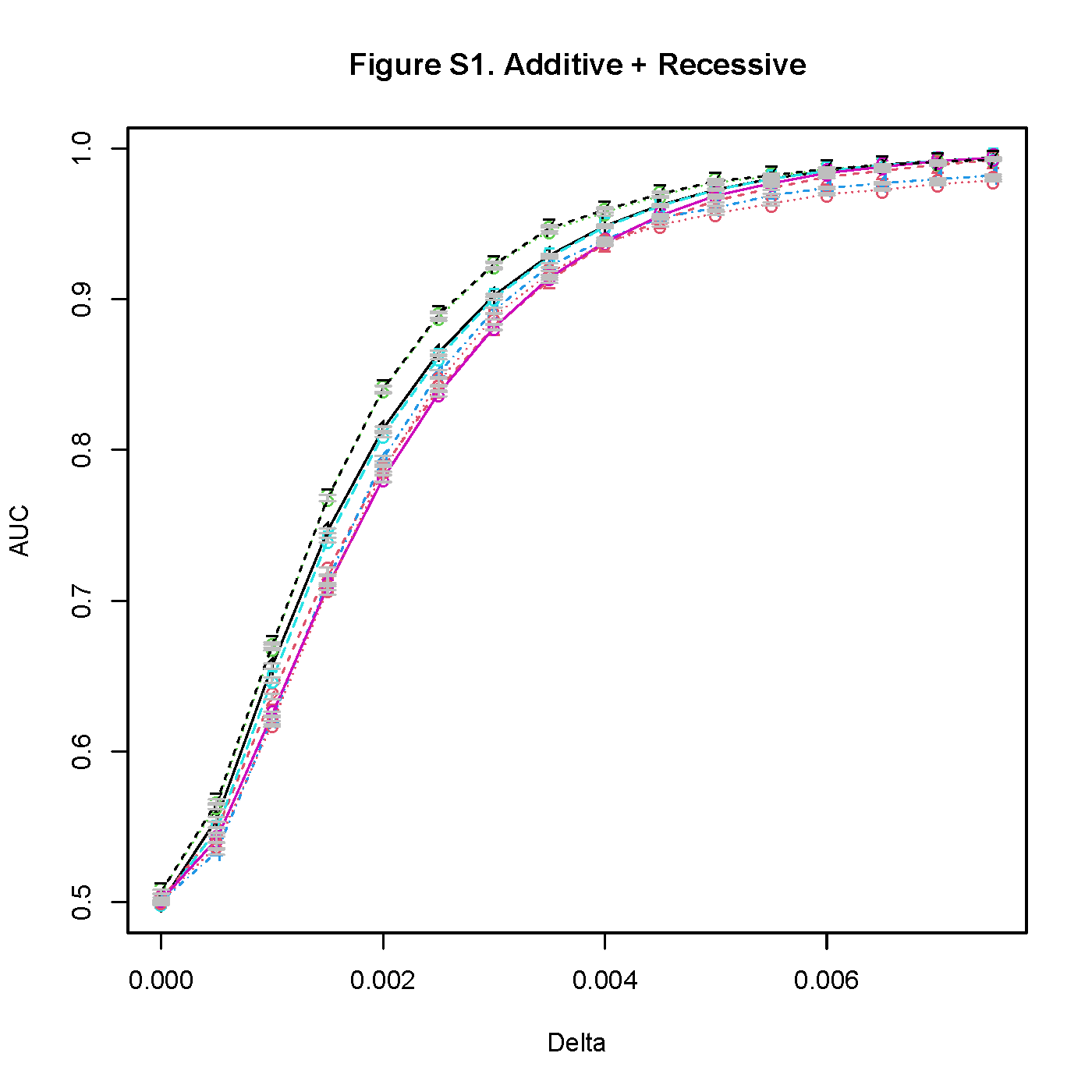

Supplement: Supplementary file 1 — S1. AUC vs. penetrance $\:\widehat{\delta\:}$ for a mixed inheritance model (400 additive, 100 recessive effect sites). Colors are the same as for Fig. 1. [file 439_2025_2798_MOESM1_ESM.tif]

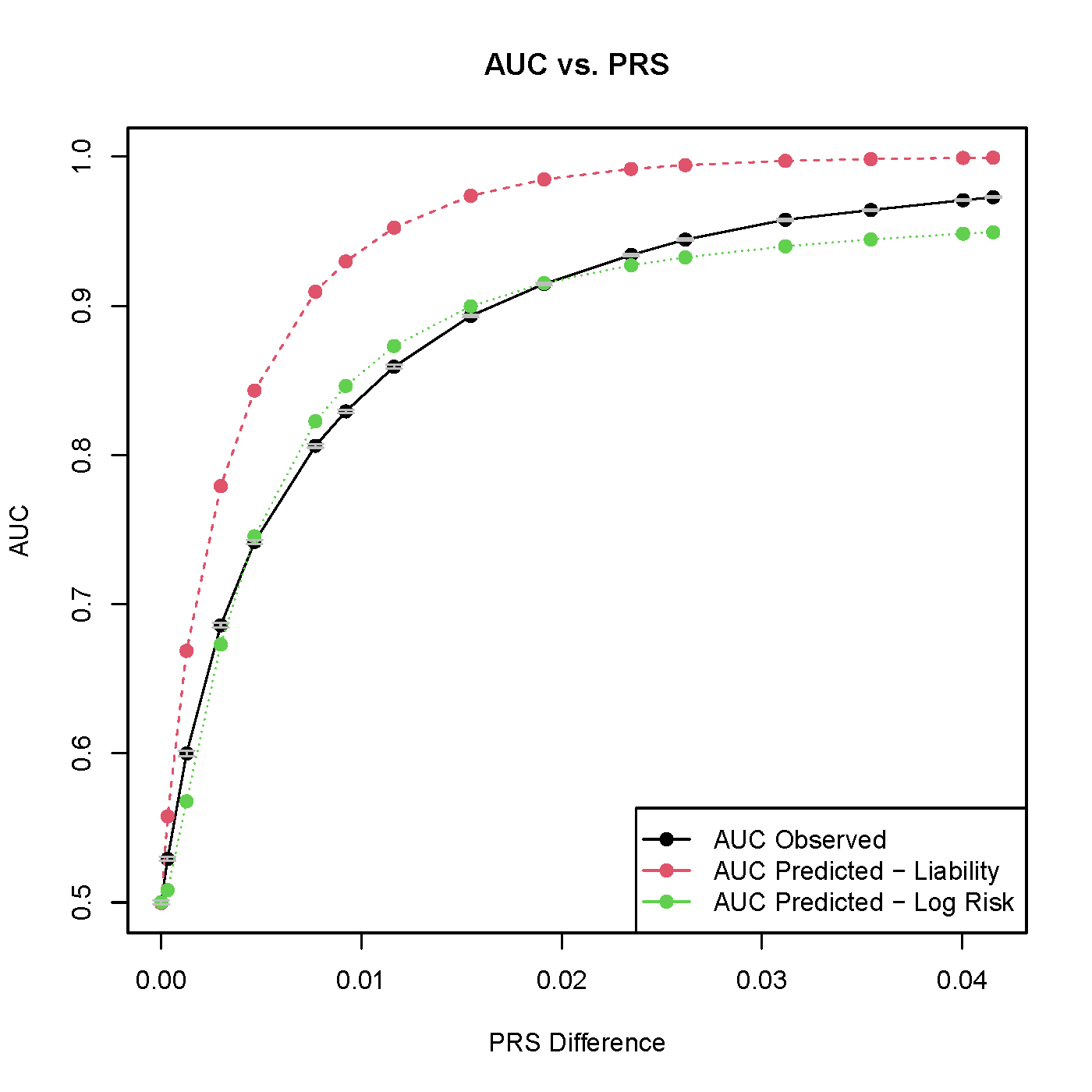

Supplement: Supplementary file 2 — S2. AUC vs. PRS difference for dominant (A) and recessive (B) models of inheritance (in black), compared to predicted values of AUC from PRS values under an additive model of inheritance. As in Fig. 3, the green line shows predicted values with a log odds risk model, the red line predicted AUC with a liability threshold model. [file 439_2025_2798_MOESM2_ESM.tif]
